# Community case study for surveillance and early case-detection of SARS-CoV-2 infections across high-risk key populations: the Sentinella programme

**DOI:** 10.3389/fpubh.2024.1432157

**Published:** 2024-10-23

**Authors:** Maela Tebon, Ruth Joanna Davis, Alessia Savoldi, Nicola Soriolo, Sarah Elizabeth Jane Walters, Michela Nosè, Corrado Barbui, Giulia Turrini, Elisa Danese, Giuseppe Lippi, Riccardo Cecchetto, Annarita Mazzariol, Davide Gibellini, Gulser Caliskan, Pierpaolo Marchetti, Giuseppe Verlato, Andrea Princivalle, Stefano Porru, Evelina Tacconelli, Pasquale De Nardo

**Affiliations:** ^1^Division of Infectious Diseases, Department of Diagnostics and Public Health, University of Verona, Verona, Italy; ^2^WHO Collaborating Centre for Research and Training in Mental Health and Service Evaluation, Department of Neuroscience, Biomedicine and Movement Sciences, Section of Psychiatry, University of Verona, Verona, Italy; ^3^Section of Clinical Biochemistry, Department of Neurosciences, Biomedicine and Movement Sciences, University of Verona, Verona, Italy; ^4^Microbiology Section, Department of Diagnostics and Public Health, University of Verona, Verona, Italy; ^5^Unit of Epidemiology and Medical Statistics, Department of Public Health and Community Medicine, University of Verona, Verona, Italy; ^6^Section of Occupational Medicine, Department of Diagnostics and Public Health, University of Verona and Clinical Unit of Occupational Medicine, Integrated University Hospital of Verona, Verona, Italy

**Keywords:** COVID-19, community-engagement, surveillance, outbreak, public health emergency

## Abstract

At the beginning of the COVID-19 pandemic, an *ad hoc* organisational framework was established between academic, local government and community partners to implement the “Sentinella – Identify, Trace and Prevent” screening programme in Verona, north-east Italy. Between September 2020 and May 2021, key populations not covered by any screening policies at the local and national level were screened for SARS-CoV-2. Target populations were: older adult residents (males >65 years and females >75 years), bus and taxi drivers, social workers, supermarket employees, hospital cleaning and catering staff, researchers working in the local hospitals, students, and people experiencing homelessness (PEH). Five dedicated swab clinics, home testing facilities, and one mobile clinic were activated to collect nasopharyngeal swabs. Molecular analysis was performed for all the subjects; an antigen-rapid diagnostic test (Ag-RDT) was also implemented as a point-of-care test for PEH. Medical follow-up, psychological support, and quarantine facilities were organised for subjects who tested positive for SARS-CoV-2. Overall, 2075 subjects participated in the surveillance programme. Amongst these, 1,572 were residents/workers, whilst 503 were PEH. A total of 127 (6.2%) participants tested positive for SARS-CoV-2. Sixty-nine were residents, 58 PEH. The incidence rate was 4 per 10.000 person/day (95% CI 3.1–5.0). The highest prevalence and incidence rates were found amongst supermarket employees (9.7% and 8.5 per 10.000 person/day, 95% CI 3.81–18.86, respectively), followed by hospital cleaning staff (8.1%, 7.6 per 10.000 person/day, CI 95% 4.9–11.7). Regarding PEH, the prevalence of SARS-CoV-2 was 11.5%. All PEH identified as positive were isolated in dedicated shelter facilities. Amongst the 69 residents/workers who were quarantined, 53 were reached for initial psychological support for assessing the presence of any psychological distress or psychiatric pathology. Amongst the subjects evaluated, 10 (18.9%) presented clinically significant psychological discomfort and accessed the stepped-care psychological intervention. The community partnerships played a pivotal role in optimising early case detection. Promotion of testing helped to prevent and contain more efficiently potential clusters through strategic planning, especially for PEH. Insights from the study highlight the importance of community partnerships in public health emergencies, particularly in the context of highly transmissible diseases pathways.

## Introduction

In Italy, the first locally contracted case of SARS-CoV-2 was detected at the end of February 2020 (1), and despite the implementation of restrictive measures at the local level to contain the first clusters identified in the north-east of the country, the national health system quickly became overwhelmed due to the high number of COVID-19 cases that required hospitalisation with 3,200 COVID-19 related deaths in less than 1 month (2, 3). A national lockdown with restrictive “stay home” policies was implemented at the beginning of March 2020, which led to a decrease in the incidence and, consequently, a reduction in mortality and hospitalisation (4). After a partial relief during the summer, an increase in incidence was observed in September 2020, where there was an exponential increase in cases and the commencement of the second wave (5). The Italian government gradually established public health measures, which included the obligation to wear masks in indoor and outdoor spaces, teleworking for some professional categories, as well as a surveillance programme to identify and isolate positive cases together with a contact tracing programme. Moreover, based on the regional parameters, such as Rt calculated daily, four areas of risk scenario were identified for which modular measures were implemented based on the local epidemiology (6). In particular, following the recognition of the magnitude of asymptomatic COVID-19 cases thanks to the largescale surveys performed in the town of Vo’ (7), the Veneto region put in place measures aimed at tracking the spread of the disease in real-time by implementing widespread testing programmes amongst the general population (8). The screening was extended to the older adult in nursing homes and healthcare providers (HCP), to symptomatic people and their secondary contacts, as well as to students attending primary and secondary schools (9).

In parallel to the above-mentioned regional screening programmes, the municipality of Verona launched a surveillance testing programme entitled “Sentinella: Identify, Trace and Prevent” targeting specific categories who were not covered by existing screening policies. The Sentinella programme was implemented from September 2020 to May 2021 and adopted a community engagement approach that responded to the regional programming of phase 3 of the DGPR334/2020, involving local community and government stakeholders. Indeed, the role of the community as a key partner in public health emergencies is well established in the literature and is particularly relevant in the context of the highly transmissible spread that characterised the COVID-19 pandemic. With this Community Case Study, we highlight how different actors – including religious organisations, local businesses and local service providers - came together each from a different perspective, and in the absence of a pre-existing cooperation framework, to successfully implement a surveillance plan during a public health emergency as was the COVID-19 pandemic.

## Methodology

### Setting and population

**Figure 1:**
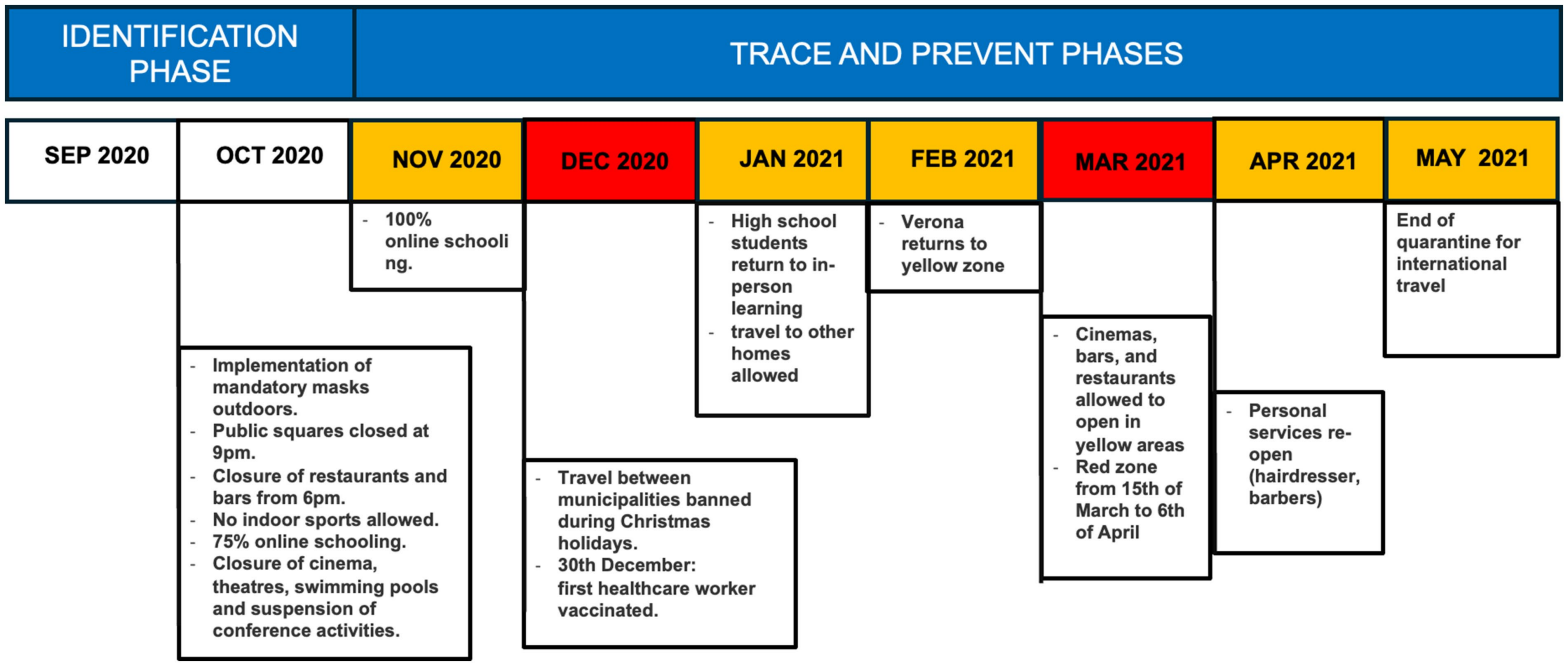
Overview of the timeline of the restrictions in conjunction with the implementation of the Sentinella project.

The surveillance programme was conducted in the municipality of Verona, an industrialised city with a high standard of healthcare located in the north-east of the Veneto region in Italy, with a population of approximately 255,000 residents (10). At the beginning of September 2020, Verona municipality had experienced a total of 6,500 cases and 600 deaths since the beginning of the pandemic (11), with cases increasing daily. The period of the surveillance (September 2020 to May 2021) also coincided with the reintroduction of restrictions and health measures at regional level (Figure 1).

The target population was selected on the basis of the assessment of target groups already included in periodic SARS-CoV-2 screenings planned at regional level, adopting the following criteria: increased risk of severity and mortality according to epidemiological data, increased risk of transmission based on work activities and workplace, and higher probability to acquire SARS-CoV-2 infection due to the environmental conditions. The evidence indicated that patients >65 years with comorbidities were at increased risk of death (12), with males having an increased risk of mortality compared to females (13). For this reason, males aged over 65 years and females aged over 75 years were selected as a target group. In relation to the professional groups, preliminary evidence (14, 15) suggested specific categories of the population at increased risk of infection, such as public transport workers (bus drivers, taxi drivers) and supermarket employees. Hospital cleaning and catering staff and researchers working at the hospital were also considered at higher risk of infection due to the workplace. Based on the evidence of several environmental factors involved in COVID-19 transmission, including enclosed spaces with inadequate ventilation and crowded contact settings, also students in shared university accommodation and individuals living in shelters were identified as target groups. In particular, the Verona municipality implements a cold-weather emergency plan during the winter to provide accommodation to people living in extreme fragility at the available shelters. Given that people experiencing homelessness (PEH) were identified as being particularly vulnerable to severe SARS-CoV-2 infection (16, 17), all individuals ≥18 years requesting a temporary residence at shelters run by the religious organisation CARITAS were screened for SARS-CoV-2 infection regardless of the presence of symptoms. The distribution of each group is summarised in Table 1.

**Table 1:** Sentinella target population.

Sentinella target population Total	*N* (%) 2075
People experiencing homeless (PHE)	503 (24.2)
Male >65 and female >75	310 (14.9)
Hospital research staff	260 (12.5)
Hospital cleaning staff	246 (11.9)
Public bus drivers	199 (9.6)
Social workers	181 (8.7)
Students sharing residences	138 (6.7)
Fast food, beauticians, hairdresser	74 (3.6)
Supermarket employees	62 (3.0)
Hospital catering staff	59 (2.8)
Taxi drivers	43 (2.1)

### The Sentinella community partnership

The Sentinella programme “Identify, Trace and Prevent” was conceived, designed and implemented through a collaboration between the University of Verona (UNIVR), the municipality of Verona, the local hospital administration Azienda Ospedaliera di Verona (AOUI-VR), the local public health authority (ULSS 9), a local religious organisation (CARITAS diocesana), local businesses (Azienda Trasporti Verona - ATV, Migross, Esselunga, local beauticians and hairdressers) and a student representative body (European Students Union, ESU). This was the first time that these actors had come together under the umbrella of a formal network to respond to a public health emergency.

UNIVR was responsible for reviewing the literature and developing the protocol for the implementation of the activities and for the overall coordination of the programme. Multiple departments from UNIVR were involved, namely Infectious Diseases, Microbiology and Clinical Biochemistry, Psychiatry, Statistics, and Occupational Medicine.

The municipality of Verona, as the representative body for citizens, was in charge of leading the outreach programme, organising the communication activities through direct invitations, outlining the objectives of the programme and providing the addresses of the testing locations, links to dedicated websites, and useful contacts. The municipality was also responsible for the organisation of community meetings and in collaboration with CARITAS diocesana, coordinated the setting up of the additional clinics for testing as well as the identification of temporary accommodation to isolate positive PEH.

Communication activities were also supported by a local media agency (Athesis editorial group) and by the stakeholders themselves (supermarket managers, directors of the local transport industry) who promoted the surveillance amongst its employees. Public transport services (buses and taxis), supermarkets and other employment categories (hospital cleaning and catering staff) and the students union were involved as categories of target populations, contributing to the consultation and feedback phases of the programme.

The Local Public Health authority (ULSS 9) was involved in contact tracing once alerted to a positive swab through the regional monitoring system already in place, into which the Sentinella programme was automatically connected.

**Figure 2:**
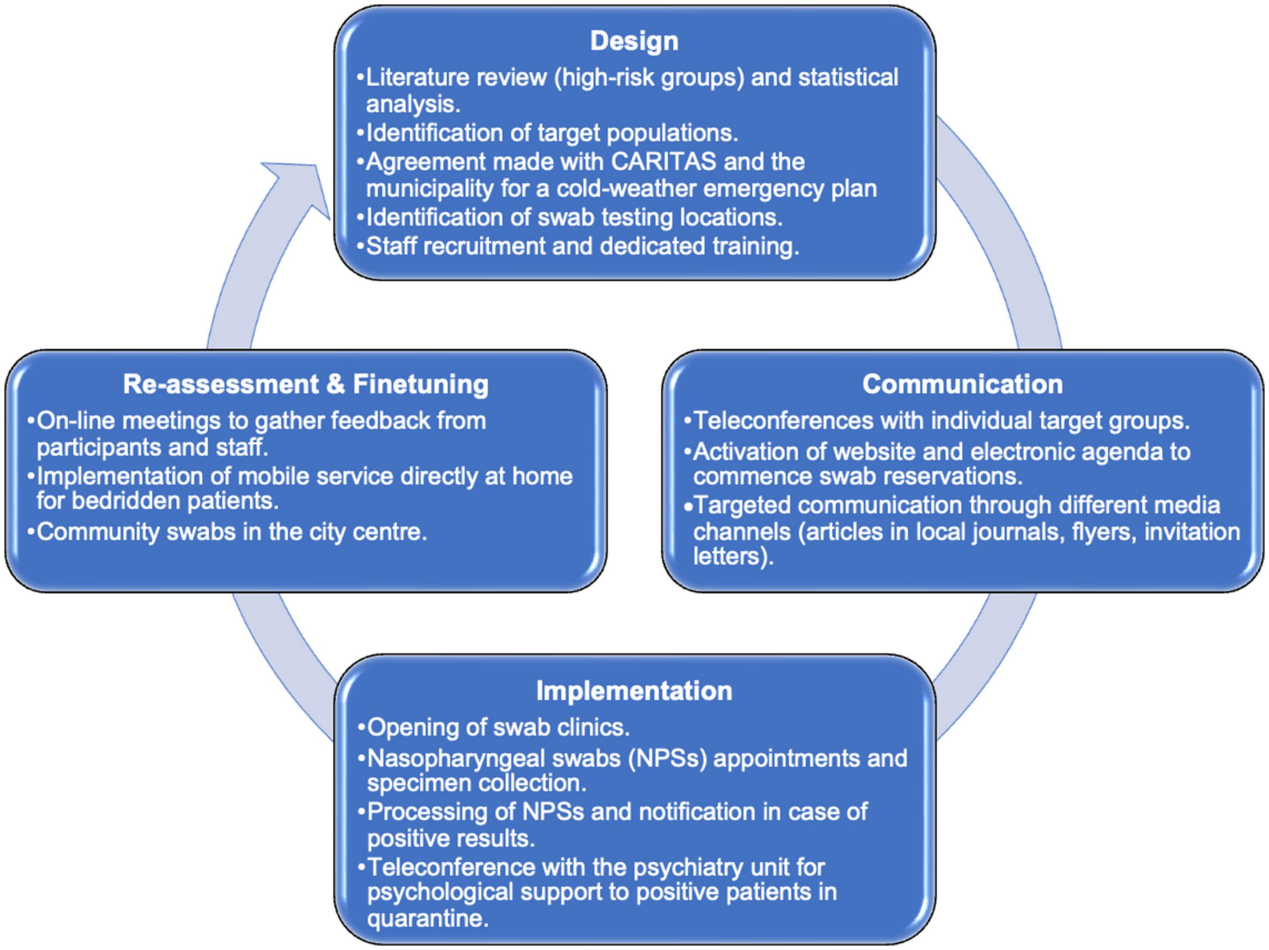
Adaptation and re-modulation during the engagement process.

For an overview of the engagement process, see Figure 2.

### Surveillance design: identify, trace and prevent

The programme was structured according to three main interventions: identification (of target groups), tracing (of potential contact and cases) and prevention (isolation and precautions). The identification phase started on 1st of September through a multidisciplinary cooperation between the main stakeholders. The Verona records office provided a list of all the older adult persons meeting the inclusion criteria. The UNIVR Statistics department sampled all eligible residents within 1.5 km of the dedicated swab clinics. Residents were then invited randomly based on the proximity to the clinics to reduce barriers to testing. With the support of the UNIVR Occupational Health Unit, the managers of selected businesses and workplaces were contacted and consulted on their willingness to be involved. During the project implementation, the additional target population of the students was included in collaboration with ESU, which informed the students via an e-mailing list.

The trace and prevention phase of the Sentinella programme lasted from the 16th of November 2020 to 28th May 2021 and included nasopharyngeal swab (NPS) and molecular analysis performed with polymerase chain reaction (PCR) tests for SARS-CoV-2, a self-administered structured questionnaire collecting general health information (comorbidities, medical history, vaccination status, and COVID-19 related-symptoms during the 3 weeks previous to the swab collection and on the day of swab collection). For the PEH subgroup, an additional NPS was collected to perform the rapid-antigen test at the point of care.

### Determining swab testing locations

Five dedicated swab clinics were set up. The location of the clinics was discussed by all the stakeholders considering the need to take advantage of existing medical infrastructures whilst at the same time facilitating the access to testing. For example, the clinic at the train station was a convenient stop during working hours for taxi drivers and close to the reception of cold-weather emergency for PEH. The clinic set up at the bus drivers’ headquarters provided bus drivers an easy access to testing before starting their morning shift. For older adult persons with mobility issues and bedridden patients, a mobile service was made available to collect the samples directly at home. The clinics were open every weekday with flexible timetables adapted to the number of requests.

### Collection and processing of samples

A dedicated on-line agenda was set up to schedule the testing of participants who were able to access it directly from the mobile phone to book the most convenient appointment. A dedicated phone-line was also active Monday to Friday with office hours to support participants in the booking process. During the first visit, a medical doctor collected a signed informed consent after explaining the aims, procedures and the timeline of the surveillance. Collection of NPS was performed by trained nurses following the standard procedure.

At the end of each day, a dedicated driver collected the NPSs from each swab clinic and delivered them to the UNIVR Microbiology department. NPSs were processed daily for the molecular detection of SARS-CoV-2. The results of NPS that were performed from Monday to Thursday were conveyed within 24–48 h, whilst swabs performed on Friday were reported within 72 h at most. Results were available in real-time on the personal electronic health record.

### Management of positive results

In the case of a positive result, the participant was informed by phone within 24 h of receiving the result through a telemedicine consultation with an infectious diseases physician to investigate the patients’ health status. Asymptomatic patients were followed up through a bi-weekly assessment until the evidence of a negative swab. In the case of the presence of symptoms, the patient was referred to the general practitioner. Moreover, psychological distress as measured by the Kessler Psychological Distress Scale (K10) and functional impairment as measured by the WHO Disability Assessment Schedule (WHODAS) were assessed in all participants, showing positive results. Participants who did not show psychological distress were offered a health promotion intervention by a psychologist, through 2–3 telematic meetings tailored to the individual’s characteristics and needs. In case of clinically significant psychological distress, a stepped-care programme consisting of two digital psychosocial interventions developed by the WHO was offered. The first step consisted of a weekly guided self-help stress management course adapted from Self Help Plus (SH+), called “Doing What matters in times of Stress (DWM),” with a psychologist support through practical exercises and key concepts over the phone. The second step was the “Problem Management Plus (PM+),” an individual five-session psychological intervention based on problem-solving and cognitive behavioural therapy techniques delivered individually through video calls and offered only to participants who continued to show elevated levels of psychological distress after step 1. In case of persisting problems, participants were referred to a physician or specialist.

**Figure 3:**
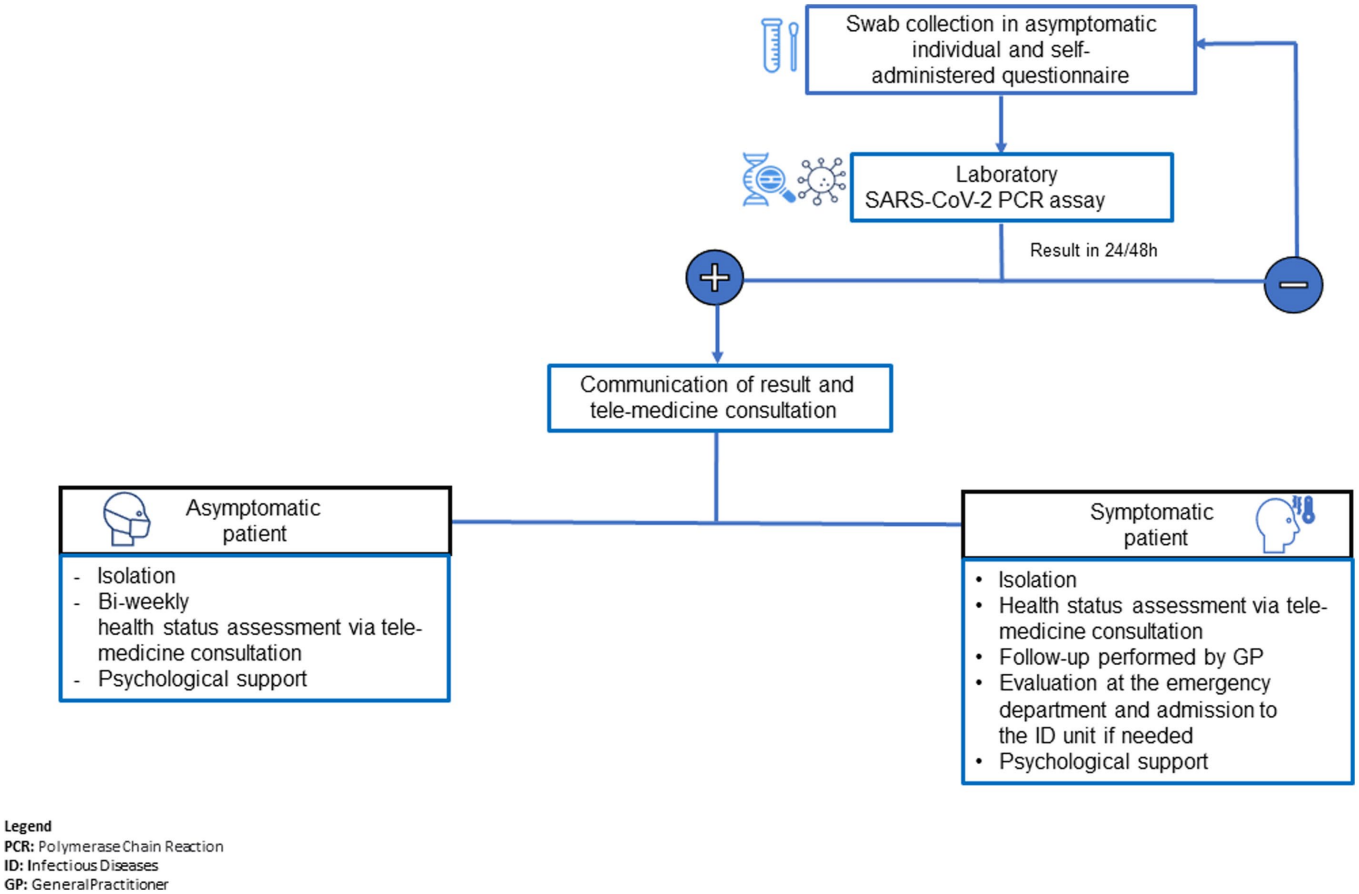
Patient management flowchart.

The patient management is summarised in the flowchart shown in Figure 3.

### A tailored approach for PEH

To adapt to the target population’s needs, a dedicated testing process was put in place for PEH. The monitoring process consisted of collecting two NPSs before admission to the shelter, one for the Ag-RDT and the other for the molecular test. In case of a negative test, the individual was allowed to access the shelter, whilst in case of a positive Ag-RDT, the subject was hosted in a dedicated isolation centre for subsequent monitoring and medical care, if needed. In case of a positive molecular test result of a subject already assigned with a bed in the shelter after testing negative to the rapid test, the medical staff was responsible for rapidly communicating the result to the subject and the shelter coordination team to implement the procedure for isolation. Support staff were present to ensure that all preventive measures like handwashing, social distancing, and wearing of face masks were respected.

### Statistical analysis

Categorical variables were summarised with percentages and continuous variables with median and interquartile range. Fisher exact test was used to assess any potential difference in prevalence of SARS-COV-2 infection across key populations. The incidence density rates (per 10,000 person/day) were obtained by dividing the number of the subjects with at least one positive NPS by the total number of person-days at risk. *p* value >0.05 was regarded as statistically significant. All analyses were conducted using STATA (STATA Corp version 16, Texas, USA).

## Results

Overall, 2075 subjects participated in the surveillance programme with 9,988 swabs processed.

Excluding the PEH group, 1,572 participants were followed up under periodic surveillance, accounting for a total of 9,256 samples processed. As shown in Table 2, the median age was 47.6 (Interquartile range, IQR, 34.0–58.9); 835 (53%) subjects were female. Males >65 and females >75 were the most represented population (310; 19.7%), followed by hospital research staff (260; 16.5%), hospital cleaning staff (246; 15.7%), and bus drivers (199, 12.7%). The mean follow-up period was 116 days per subject (standard deviation, SD ±24 days). The average number of RT-PCR performed per subject was six. The prevalence of SARS-CoV-2 infection across residents/workers was 4.4%, with a significant difference amongst sub-cohorts (*p* = 0,009). The highest prevalence was found amongst supermarket employees and hospital cleaning staff (9.7 and 8.1%, respectively) followed by hospital cleaning staff and university students sharing residences (5.1%).The majority of subjects who tested positive for SARS-CoV-2 did not report any symptoms on the day of NPS collection (44, 64%). The incidence rate was 4.0 per 10,000 person/day (95% confidence interval (CI) 3.1–5.0). The highest incidence rate was observed amongst supermarket employees (8.47, 95% CI 3.81–18.86) followed by hospital cleaning staff (7.6, CI 95% 4.9–11.7), university students sharing residences (5.5, CI 95% 2.6–11.5) and hospital catering staff (4.0, CI 95% 1.3–12.3). The incidence trend over time was almost overlapping between the population included in the Sentinella programme and general population in Verona municipality, with the former group exhibiting overall a lower incidence (Supplementary material).

**Table 2:** Prevalence rate of SARS-CoV-2 infection amongst residents/workers sub-populations.

Variables		Overall *n* = 1,572 (%)	Negative *n* = 1,503 (%)	Positive *n* = 69 (%)	*p* Value
Age (median, IQR)			47.7 (33.9–58.8)	46.8(36.2–59.1)	0.933
Male sex, *N* (%)		739 (47.0)	709 (95.9)	30 (4.1)	0.548
Resident/worker population					0.009
	Hospital research staff	260 (16.6)	254 (97.7%)	6 (2.3%)	
	Hospital cleaning staff	246 (15.6)	226 (91.9%)	**20 (8.1%)**	
	Public bus drivers	199 (12.7)	191 (96.0%)	8 (4.0%)	
	Social workers	181 (11.5)	177 (97.8%)	4 (2.2%)	
	Students sharing residences	138 (8.8)	131 (94.9%)	7 (5.1%)	
	Supermarket workers	62 (3.9)	56 (90.3%)	**6 (9.7%)**	
	Hospital catering staff	59 (3.8)	56 (94.9%)	3 (5.1%)	
	Taxi drivers	43 (2.7)	41 (95.3%)	2 (4.7%)	
	Male >65 and female >75	310 (19.7)	297 (95.8%)	13 (4.2%)	
	Fast food, beauticians, hairdresser	74 (4.7)	74 (100.0%)	0 (0%)	

IQR: interquartile range.

Amongst the 69 subjects who were in isolation, 53 were successfully contacted for initial psychological support and to assess the presence of any psychological distress or mental disorders. Amongst the subjects evaluated, 10 (19%) presented clinically significant psychological distress. Eight participants participated in the DWM stress management course. None of them showed elevated levels of psychological distress upon completion, so none underwent the PM+ intervention.

With regard to PEH cohort, 503 subjects were monitored over two winter seasons, with a total of 732 NPS processed. Fifty-eight (8%) individuals tested positive and were isolated in the dedicated shelter in order to avoid further viral spread and outbreaks in shelter facilities. Being PEH particularly exposed to SARS-CoV-2 infection, an ancillary study on the diagnostic accuracy of Ag-RDT as point-of-care test was conducted on PEH as part of the Sentinella programme with the aim of investigating the performance of this test as a screening tool compared with gold standard PCR (18).

## Discussion

### Community partnerships supporting surveillance in infectious disease outbreaks

Community engagement approaches have shown their importance through various infectious diseases outbreaks. As an example, during the Ebola emergency the active involvement of local people was crucial for enabling health interventions (19). During the first wave of the COVID-19 pandemic, East Asian countries focused their efforts on strengthening surveillance programmes in the community. Indeed, screening programmes, rigorous contact tracing process, and community-centred public health systems played a pivotal role to contain the spread of SARS-CoV-2 and to decrease the mortality rate (20–25). Gilmore et al. (20) highlighted the relevance of surveillance and contact tracing to support equity-informed response during COVID-19 prevention and control programmes. Pritchard et al., (26) by implementing a real-time large community-based survey, identified the different factors and specific behaviours driving the SARS-CoV-2 positive cases for monitoring trends across United Kingdom to inform public health policy.

The Sentinella programme was implemented in Verona, where COVID-19 had a significant impact during the first and second waves. The intervention was designed in line with the emerging scientific evidence at the time of its implementation, which underlined the importance of the identification of pauci- and asymptomatic infections to prevent future outbreaks (7). The programme was designed and conducted with the involvement of multiple community actors and through a flexible approach allowing for in-progress adaptation in response to emerging needs and feedback. Critical issues that were flagged were: the need to widen the focus of the programme from the surveillance to a more multidisciplinary approach including also psychological support and occupational safety; the need to improve access to the testing both by increasing the number of testing sites and by identifying strategic locations at the workplace, improving the flexibility of testing hours in accordance to working time, and fostering the outreach of young people through the activation of mobile screening units targeting places of social aggregation in downtown Verona.

The results demonstrated high circulation of SARS-CoV-2 in the community amongst mostly asymptomatic individuals not covered by screening programmes. Several studies on at-risk populations for acquiring SARS-COV-2 show similar results with high prevalence of infection in public workers such as bus drivers, taxi drivers, and supermarket employees (15, 27–29). The results from Sentinella further support the growing evidence of the importance of surveillance programmes for workers outside the healthcare setting during infectious disease outbreaks.

### Importance of local and contextual factors

In Italy, health systems are governed at regional level whilst during the COVID-19 pandemic certain restrictions and containment measures were adopted in accordance to national directives. The results of Sentinella programme highlight the possibility to implement a local initiative that is complementary to regional and national public health measures. Other examples have shown how urban interventions for COVID-19 response can co-exist in parallel with centrally led measures and, in some cases, even guide future strategies based on emerging results (30).

Involvement of local stakeholders, such as CARITAS, who have an in-depth knowledge of the environment and social context and an established relationship with the target population (PEH), is crucial. Insights on resident density and room organisation prompted the planning of the tailored surveillance of PEH population based on the use of Ag-RTD allowing for an accessible testing service driving prompt isolation and management of the positive individuals (18). The positive outcome of the PEH surveillance carried out in Verona during the winter 2020–2021 led to the application of the same strategy during the following winter 2021–2022. Considering the high prevalence of the positive asymptomatic PHE, we believe that the customised plan for this target subgroup successfully avoided COVID-19 outbreaks in the shelters which were not contemplated in national surveillance plans.

### Flexibility in a constantly evolving epidemiological situation

The national lockdown introduced in Italy during the first wave was deemed necessary to decrease the burden on the health care system in the absence of effective medical treatment (31). During the second wave, flexible and tailored measures were introduced through the Ministerial decree 275, intensifying tracing procedures and promptly reacting to the changes in the regional epidemiological trend.

The local management of the surveillance allowed prompt adaptation and rapid mobilisation of resources in case of additional requirements. As an example of this flexible and problem-oriented approach, the students’ population was incorporated *in itinere*, based on the COVID-19 clusters that occurred in the shared accommodation and reported by the local ESU. Moreover, the regional campaign of the “spritz hour” was implemented through the prompt coordination of the Sentinella team in recognition of the local cultural norm of young people congregating in the same place (the town centre) at the same time. This event represented an ideal opportunity to access a significant number of young people and implement both large scale testing using Ag-RDT, supplied by the Veneto region, whilst at the same time providing educational activities on prevention measures such as social distancing, mask wearing and hand-hygiene (32).

### Academia as a trusted partner in the community

The containment measures put in place during the pandemic were often regarded as coercive by the population, especially when prolonged or repeated over time, and contributed in several occasions to a general feeling of distrust toward the government. Indeed, despite the positive effects in reducing SARS-CoV-2 spread, the restrictive mandatory policies had important repercussions on the social and economic systems (33, 34) and led to a consistent deterioration of mental health of the general population (35). In public health emergencies, the establishment of collaborations and community-academic partnerships is crucial for building synergies, sharing knowledge and, ultimately, increasing the overall acceptability of the required interventions (36, 37). In the Sentinella programme, UNIVR and the Verona University Hospital were perceived by the local population as trusted partners. Moreover, the general apprehension experienced by the community toward the testing process was counterbalanced by the offer of a direct medical support and follow-up and proved to be a winning strategy in terms of recruitment.

## Limitations

Fewer older adult people were enrolled in the intervention compared to expectations, underlying the need of exploring potential organisational barriers for older adult involvement in surveillance programmes during a pandemic. The programme reported the essential elements of community engagement and partnership that can be applied, in principle, to public health emergencies represented by a pandemic. Nonetheless, given the context and the specific characteristics of the intervention, the results may not be fully generalizable to different cultures or settings.

## Conclusion

Our results provide useful insights into how partnering of local government and local public health bodies with community stakeholders can support biomedical approaches and surveillance efforts to optimise and successfully implement future control strategies in case of infectious diseases outbreaks. The involvement of local institutions can play a pivotal role in increasing the overall trust of the population as regards government decisions and acceptability of containment measures in case of health emergencies.

## Data Availability

The raw data supporting the conclusions of this article will be made available by the authors, without undue reservation.
