# Supplementary figures and images for: Community case study for surveillance and early case-detection of SARS-CoV-2 infections across high-risk key populations: the Sentinella programme
Source: Front Public Health. 2024 Oct 23;12:1432157. doi: 10.3389/fpubh.2024.1432157 (PMC11541710; doi:10.3389/fpubh.2024.1432157)

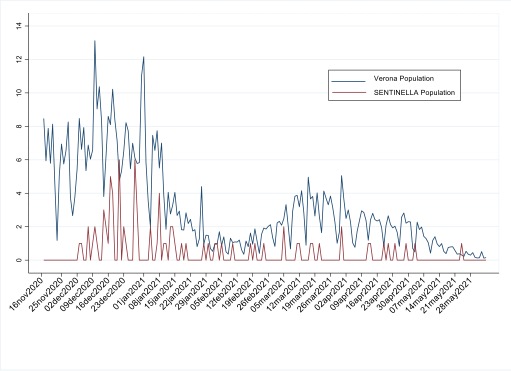

Supplement: Supplementary file 1 [file Image_1.JPEG]
